# The Mediating Role of Cortical Atrophy on the Relationship between the Resilience Index and Cognitive Function: Findings from the Healthy Brain Initiative

**DOI:** 10.1192/j.eurpsy.2025.1685

**Published:** 2025-08-26

**Authors:** R. Ezzeddine, D. Oshea, S. Camacho, L. Besser, M. Tolea, J. Galvin, C. Galvin, L. Wang, G. Gibbs

**Affiliations:** 1 Psychiatry; 2 Neurology; 3 University of Miami, Miami, United States

## Abstract

**Introduction:**

**Background:** Lifestyle factors are linked to differences in brain aging and risk for Alzheimer’s disease, underscored by concepts like ‘cognitive reserve’ and ‘brain maintenance’. The Resilience Index (RI), a composite of 6 factors (cognitive reserve, physical and cognitive activities, social engagement, diet, and mindfulness) provides such a holistic measure.

**Objectives:**

This study aims to examine the association of RI scores with cognitive function and assess the mediating role of cortical atrophy.

**Methods:**

Baseline data from 113 participants (aged 45+, 68% female) from the Healthy Brain Initiative were included. Life course resilience was estimated with the RI, cognitive performance with Cognivue®, and brain health using a machine learning derived Cortical Atrophy Score (CAS). Mediation analysis probed the relationship between RI, cognitive outcomes, and cortical atrophy.

**Results:**

In age and sex adjusted models, the RI was significantly associated with CAS (β= -0.25, p = 0.006) and Cognivue® scores (β= 0.32, p < 0.001). The RI-Cognivue® association was partially mediated by CAS (β= 0.07; 95% CI [0.02, 0.14]).

**Conclusions:**

Findings revealed that the collective effect of early and late-life lifestyle resilience factors on cognition are partially explained by their association with less brain atrophy. These findings underscore the value of comprehensive lifestyle assessments in understanding the risk and progression of cognitive decline and Alzheimer’s disease in an aging population.

**Disclosure of Interest:**

R. Ezzeddine: None Declared, D. Oshea: None Declared, S. Camacho: None Declared, L. Besser: None Declared, M. Tolea: None Declared, J. Galvin Employee of: Cognivue (Chief Scientific Officer of Cognivue). Cognivue devices are used for research conducted at the center., C. Galvin: None Declared, L. Wang: None Declared, G. Gibbs: None Declared

